# *Cyatta abscondita*: Taxonomy, Evolution, and Natural History of a New Fungus-Farming Ant Genus from Brazil

**DOI:** 10.1371/journal.pone.0080498

**Published:** 2013-11-15

**Authors:** Jeffrey Sosa-Calvo, Ted R. Schultz, Carlos R. F. Brandão, Christiana Klingenberg, Rodrigo M. Feitosa, Christian Rabeling, Maurício Bacci, Cauê T. Lopes, Heraldo L. Vasconcelos

**Affiliations:** 1 Maryland Center for Systematic Entomology, Department of Entomology, University of Maryland, College Park, Maryland, United States of America; 2 Department of Entomology, National Museum of Natural History, SmithsonianInstitution, Washington, District of Columbia, United States of America; 3 Museu de Zoologia, Universidade de São Paulo, São Paulo, São Paulo, Brazil; 4 Abteilung Entomologie, Staatliches Museum für Naturkunde, Karlsruhe, Karlsruhe, Germany; 5 Departamento de Zoologia, Universidade Federal do Paraná, Curitiba, Paraná, Brazil; 6 Museum of Comparative Zoology, Harvard University, Cambridge, Massachusetts, United States of America, United States of America; 7 Centro de Estudos de Insetos Sociais, Universidade Estadual Paulista, Rio Claro, São Paulo, Brazil; 8 Instituto de Biologia, Universidade Federal de Uberlândia, Uberlândia, Minas Gerais, Brazil; Onderstepoort Veterinary Institute, South Africa

## Abstract

*Cyatta abscondita*, a new genus and species of fungus-farming ant from Brazil, is described based on morphological study of more than 20 workers, two dealate gynes, one male, and two larvae. Ecological field data are summarized, including natural history, nest architecture, and foraging behavior. Phylogenetic analyses of DNA sequence data from four nuclear genes indicate that *Cyatta abscondita* is the distant sister taxon of the genus *Kalathomyrmex*, and that together they comprise the sister group of the remaining neoattine ants, an informal clade that includes the conspicuous and well-known leaf-cutter ants. Morphologically, *Cyatta abscondita* shares very few obvious character states with *Kalathomyrmex*. It does, however, possess a number of striking morphological features unique within the fungus-farming tribe Attini. It also shares morphological character states with taxa that span the ancestral node of the Attini. The morphology, behavior, and other biological characters of *Cyatta abscondita* are potentially informative about plesiomorphic character states within the fungus-farming ants and about the early evolution of ant agriculture.

## Introduction

New insect species are being discovered at a rate that is at least twice the historical average [1–3]. As we might expect, many newly discovered species are cryptic species (i.e., multiple species previously thought to comprise a single species) and/or are easily referable to well-known genera or species groups. A significant number of newly discovered species, however, are phylogenetically important, i.e., they are the sole representatives of previously unknown, anciently diverged lineages and, as such, sources of new information about early insect evolution. New supraspecific taxa (e.g., new genera, families, etc.) are commonly erected for such species. For example, a new order of insects, the Mantophasmatodea, was described as recently as 2002 [4–8] (currently considered a suborder of *Notoptera* [9]). 

Despite their status as a relatively well-studied group, ants (Formicidae) are no exception to this trend of increasing species discovery [10]. Over 12,700 ant species in 308 genera have so far been described [11,12], yet most ant systematists estimate that there are twice as many extant species [13,14], which would make ants the most speciose family of social insects [12]. In recent years, more than 19 new extant and *ca.* 17 extinct ant genera and one subfamily (Martialinae) have been described [15–36].

Fungus-farming "attine" ants are exclusively New World in distribution, ranging from the United States in the North to Argentina in the South [37–39] with six genera (*Acromyrmex, Atta, Cyphomyrmex, Mycetophylax, Mycocepurus*, and *Trachymyrmex*) also present in the Caribbean [30,39,40]. To date, 256 valid species have been described in 15 extant genera and in one ichnogenus (*Attaichnus* Laza) [11,30]. Because attine ants participate in complex associations with their cultivated fungi and other microbial symbionts, they have become model systems for the study of symbiosis and coevolution [41–61]. Attine ants have additionally become model systems for comparative evolutionary studies of mating frequency/polyandry, gyne number, parthenogenesis, social parasitism, caste polyethism and polymorphism, nesting behavior, foraging behavior, and diverse microbial symbioses [47,48,57,62–70]. Leaf-cutter ants in particular are the subjects of a century of applied research due to their status as serious pests of agriculture in Central and South America [71–85].

Here we describe the sole representative species of a new genus, *Cyatta abscondita* gen n. et **sp n.**, in the fungus-farming ant tribe Attini (Formicidae: Myrmicinae) and document its presence in the Brazilian Cerrado, a global biodiversity hotspot [86]; in the Caatinga, a xeric shrub-land and thorn forest in northeastern Brazil; and in Atlantic semi-deciduous forest, considered a transitional zone between humid Atlantic forests and the drier biomes of the Caatinga and Cerrado [87]. We describe the morphology, behavior, fungal associations, nest architecture, and other biological characters of *C. abscondita* that are potentially informative about plesiomorphic character states within the tribe Attini and within the informal clade Neoattini and, consequently, about the early evolution of ant farming behavior. 

## Materials and Methods

### Material examined

The specimens examined have been deposited in the following institutions:

BLME Coleção Entomológica, Bacci Laboratory of Molecular Evolution, São Paulo State University (UNESP), Rio Claro, Brazil.

CRC C. Rabeling Collection, Cambridge, MA, U.S.A.

DZUP Coleção Entomológica “Pe. Jesus Santiago Moure”, Departamento de Zoologia, Universidade Federal do Paraná, Curitiba, PR, Brazil.

MCZ Museum of Comparative Zoology, Harvard University, Cambridge, MA, U.S.A.

MZSP Museu de Zoologia, Universidade de São Paulo, São Paulo, Brazil.

MBC–UFU Museu de Biodiversidade do Cerrado, Universidade Federal de Uberlândia, Uberlândia, Minas Gerais, Brazil.

USNM United States National Museum of Natural History, Washington, DC, U.S.A. 

### Morphological measurements and specimen preparation

All measurements were taken to the nearest 0.001 mm and, unless otherwise noted, are in millimeters. Images of worker and gyne were generated at the USNM Ant Lab using a JVC KY–F75U digital camera mounted on a Leica Z16 APO steromicroscope attached to a Dell Optiplex GX620 computer. Composite images were assembled using Auto-Montage Pro® (Version 5.03.0061 BETA) software (Synoptics Ltd.). Images of the male were generated at the MCZ using a Leica DFC 420 digital camera mounted on a Leica MZ16 dissecting scope. Composite images were assembled using Leica Application Suite (Version 4.0) and Helicon Focus (Version 5.3) software packages. The only two larvae collected were dehydrated sequentially through a series of ethanol concentrations to 100% absolute and then critical-point dried in a Balzers CPD–030 using liquid CO_2_ at the Scanning Electron Microscopy (SEM) Lab in the SI–NMNH. Once the ethanol was replaced with CO_2_ the samples were slowly heated to the critical point, slowly depressurized back to atmospheric pressure, dried, and mounted on aluminum stubs. The two prepared larvae and an adult worker *Paratype* were sputter-coated with 60:40 wt% Gold:Palladium alloy on a Cressington Scientific 108 auto/SE sputter coater to a thickness of 20–25 nm. Scanning Electron Micrographs (SEMs) of these specimens were generated using a Philips XL–30 ESEM with Lanthanum Hexaboride (LaB6) source and with a backscatter detector. All images were cropped and edited using Photoshop CS5® (Version 12.0) (Adobe Inc.). 

The measurements, indices, abbreviations, and morphological terminology utilized throughout follow Gauld & Bolton [88], Klingenberg & Brandão [30], Rabeling et al. [89],, Serna & Mackay [90], and Sosa-Calvo & Schultz [91] and literature cited therein, with modifications where noted. Characters and terminology used in the description of the larvae are based on Schultz & Meier [92]. The following abbreviations are used in the description: w= worker, dg= dealate gyne, m= male. 

 Latitude and longitude coordinates were converted to decimal degree when needed by using the Earth Point Web Site (http://www.earthpoint.us/Convert.aspx). In cases where coordinates were not documented in the specimen label, the coordinates were estimated using Google Earth v7.0 (http://www.google.com/earth/index.html) and are presented within brackets. The distribution map of *Cyatta abscondita* was generated using the software ArcGIS v10.1 (Esri, Redlands, CA). 

### Field observations and nest excavations

Field work was conducted at Fazenda Água Limpa from 20–27 February 2009 (JSC, TRS), 12–18 April 2010 (JSC, TRS, CTL), and 14–22 September 2011 (JSC, TRS, CTL); and at the Broa preserve from 21–28 July 2011 (CR, MB). Fazenda Água Limpa (FAL) is a 4490 ha experimental farm and conservation area of the Universidade de Brasília located at S15.94938° W47.93567°, ~30 km from Brasília, DF, Brazil, at an altitude of 1048–1160 m. The Broa preserve, one of the southernmost Cerrado preserves of São Paulo state, is located at S22.18517° W47.87754°, 9.5 km northwest of Itirapina in São Paulo state, Brazil, at an altitude of 530 m. 

At FAL, nest entrances were located by following foragers carrying bait (Cream of Rice cereal liberally distributed on the ground) as they returned to the nest. Nests were excavated by first digging a trench about 1 meter wide and 1.5 meters deep and located about 1 meter from the nest entrance, and then by carefully shaving away soil in the direction of the nest entrance until either tunnels or nest chambers were encountered; see 93,94 for a detailed description of the methodology. During the course of the excavation, the trench was deepened as necessary and the dimensions, depths, and relative positions of tunnels and chambers were measured, photographed, and sketched. Fungus gardens were transferred from subterranean chambers to plastic containers with flame-sterilized spoons, knives, and/or forceps. Colonies were maintained in live culture in plastic containers, the bottoms of which were lined with plaster saturated with water. At Broa, nest contents were preserved in 95% ethanol after a few days, once the ants had reconstituted and restabilized their fungus gardens and removed all soil particles. Two FAL nests (JSC110920-01 and JSC110919-02) were maintained in live culture for up to four months (nest 4, see Table 1).

**Table 1:** Colony demographies and nest measurements of six excavated *Cyatta abscondita* nests, including depths and dimensions of individual chambers, chamber contents, fungus garden morphology, ant demographics, and additional natural history information.

						CHAMBER DIMENSIONS (cm)			
Nest	Locality	Collection code	Date	Chamber number	Depth (cm)	Height	Width	Depth	Demography, garde morphology, notes
1	FAL	JSC100412-01	April 12-18, 2010	1	70	1.2	3	N/A	2 pendant garden filaments
				2	70	1.5	2.5	N/A	empty; 3 polydesmid millipedes
				3	~75	2.5	5	5	24 pendant garden filaments
				4	80	2.5	3.5	N/A	pendant garden; 8 workers (1 callow)
2	FAL	JSC100416-01	April 17-18, 2010	1	40	0.5	1	N/A	small pendant garden; no ants
				2	46	1.5	2.5	N/A	small pendant garden; 2 workers
				3	50	0.5	1	N/A	empty
				4	55	2	3	N/A	small pendant garden; 2 workers
				5	56	2.6	3	N/A	pendant garden; 2 workers
				6	61	2.5	5.5	N/A	empty
				7	71	2.5	3	N/A	pendant garden; some workers; **dealate gyne**
3	FAL	JSC110919-02	September 19, 2011	1	29	1.5	5.5	1.3	empty
				2	35	2	3	1.2	pendant garden; 1 workers
				3	44	1.5	2	0.5	small pendant garden; a couple of workers; **dealate gyne**
4	FAL	JSC110920-01	September 20, 2011	1	38	2	3	1	small pendant garden
				2	42	3	2	2	2 workers
				3	43.5	2	2.5	3	2 workers
				4	46	2	3	1.5	15-20 pendant garden filaments
				5	50	1.5	1.5	1.5	small pendant garden; 2 workers
				6	59.5	1.5	3.5	1.2	large pendant garden; 7 workers
				7	82	1.8	2.5	3	large pendant garden; 4 workers
				8	104	1.9	4	3.5	large pendant garden; several workers; **larvae; dealate gyne**
5	Broa	CR110721-05	July 21, 2011	1	52	1.5	3	N/A	fungus garden; 13 workers
		CR110721-04		2	80	1.5	5	N/A	~50 pendant garden filaments; 10 workers; **1 male**
		CR110721-08		3	130	1	2.5	N/A	fungus garden; 3 workers
		CR110721-07		4	160	?	?	?	fungus garden; no ants; chamber collapsed during excavation
6	Broa	CR110726-09	July 26-28, 2011	1	102	?	?	?	fungus garden; 3 workers; chamber collapsed during excavation
		CR110728-01		2	195	1	3	N/A	fungus garden; 4 workers

For each nest, chambers are arranged according to depth, descending from the shallowest to deepest chambers. Chamber height refers to the chamber's vertical axis, chamber width to the horizontal axis parallel to the plane of excavation, and chamber depth to the horizontal axis perpendicular to the plane of excavation. FAL: fazenda Água Limpa, Brasília; Broa: Broa preserve, Itirapina; see text for more details.

Prior to 2009, only four isolated specimens had turned up in mass-collected ant samples. These include, from a leaf-litter sample, one specimen from riparian forest or Cerradão at Fazenda Olho D’água [611m; S18.90628° W45.53527°], Paineiras, Minas Gerais (MG), collected in 1999 and, from pitfall trap samples, one specimen from Caatinga at Reserva Particular do Patrimônio Natural (RPPN) Serra das Almas [330m; S05.16479° W40.67978°], Crateús, Ceará (CE), in 2003 and two specimens from Cerrado *sensu stricto* from the Reserva Ecológica do Instituto Brasileiro de Geografia e Estatística (IBGE) (1100m; S15.85000° W48.05000°), DF, Brasília, in 2008. More recently, in 2011, four additional stray specimens were taken in pitfall traps in Cerrado *sensu stricto* at Reserva Particular de Patrimônio Natural (RPPN) do Acangau (671m; S17.1790833° W 47.0583056°), Paracatu, Minas Gerais (MG), and in fragments of semideciduous forest at Fazenda Águas Claras (494m; S21.4023° W48.6873°), Pindorama, São Paulo (SP), and at Estação Experimental (414m; S21.5222° W49.3013°), Sales, (SP).

### Permits

Research and export permits for field work by JSC, TRS, CTL, and HLV were issued by the Conselho Nacional de Desenvolvimento Científico e Tecnológico (CNPq; Portarias No. 267, 359) and the Instituto Brasileiro do Meio Ambiente e dos Recursos Naturais Renováveis (IBAMA; permit numbers 14789–1, 147892, 14789–3). Dr. Yves P. Quinet and Francyregis Nunes allowed CRFB and RMF to study the only known specimen from the Caatinga and supported them during field work in Crateús. Prof. José Mauro da Silva Diogo, Director, allowed JSC, TRS, and CTL to work at FAL. The Fundação Acangau permitted HLV to collect at RPPN do Acangau.

### Molecular phylogenetics

DNA extraction, amplification, and sequencing were carried out at the Laboratories of Analytical Biology (LAB) at the National Museum of Natural History, Smithsonian Institution, Washington, DC. Ant genomic DNA was extracted from a worker collected at FAL, Brasília using the Qiagen DNEasy Tissue Kit. Four nuclear protein-coding genes, elongation factor 1-alpha paralog F1 (EF1α F1), elongation factor 1-alpha paralog F2 (EF1α F2), wingless (wg), and long wavelength rhodopsin (LW Rh) were amplified and sequenced following methods outlined in previous studies [95,96]. Sequences are deposited in GenBank as accession numbers KF569882-KF569887.

DNA sequences, consisting of ~2.5 kbp, were added to the aligned data set of Schultz & Brady [95] and aligned by eye in MacClade 4.08 [97]. Data were partitioned and modeled as in Schultz & Brady [95] and analyzed using MrBayes 3.1.2 [98] with two independent runs of 10 million generations, each distributed over eight chains (seven heated and one cold; temperature parameter 0.05). To avoid known problems with branch-length estimation [99], [100], branch length priors were shortened as follows: *prset* applyto = *(all*) brlenspr *= unconstrained:exponential* (100). For each partition, we applied moderately informative Dirichlet priors to the rate multipliers. Burn-in and stationarity were assessed by comparing the mean and variance of log likelihoods, both by eye and by using the Bayes Factor comparison in Tracer 1.5.0 [101]; by examination of the MrBayes ".stat" output file; and by examination of the split frequencies diagnostic. Based on this information, burn-in was set at 1 million generations. 

### Divergence time estimation

The time of divergence of the new genus *Cyatta* was estimated using the Bayesian uncorrelated lognormal approach as implemented in the program BEAST v1.7.5 [102] using a normal prior distribution for the root age (as described in Schultz & Brady [95]). Beast XML files were generated using the complementary program BEAUti v1.7.4 (as implemented in the BEAST package). The results combine two runs of 100 million generations each. Burn-in, convergence, and mixing were assessed by examining time series plots and ESS values in Tracer 1.5.0 [101]. Based on this information, burn-in was set at 20 million generations for each run.

### Nomenclatural acts

The electronic edition of this article conforms to the requirements of the amended International Code of Zoological Nomenclature ([103]), and hence the new names contained herein are available under that Code from the electronic edition of this article. This published work and the nomenclatural acts it contains have been registered in ZooBank, the online registration system for the ICZN. The ZooBank LSIDs (Life Science Identifiers) can be resolved and the associated information viewed through any standard web browser by appending the LSID to the prefix "http://zoobank.org/". The LSID for this publication is: urn:lsid:zoobank.org:pub:1CF00B3D-CE25-456A-8D12-6DDA628BC110. The electronic edition of this work was published in a journal with an ISSN, and has been archived and is available from the following digital repositories: LOCKSS [http://www.lockss.org] and PubMed Central [http://www.ncbi.nlm.nih.gov/pmc].

## Results

### Taxonomy

#### *Cyatta* gen. n

urn:lsid:zoobank.org:act:0ED4A047-327E-482C-A6AF-E92136DB5697 

Figures 1–4.

#### Type species

*Cyatta abscondita* sp. n., by present designation.

#### Worker

Small, monomorphic attine ant, total length (TL)= 2.29–2.56; Weber’s length (WL)= 0.58–0.65. Color pale yellow to light brown. Body densely reticulate and covered with minute simple appressed hairs, more abundant on dorsum of head, waist segments, and gaster than on mesosoma. Palp formula 4,2. Anterior margin of clypeus produced into a convex, almost triangular, smooth, shining flange, i.e., "clypeal apron," with long unpaired median seta that originates closer to its posterior margin. Psammophore absent. Masticatory margin of mandibles 4-toothed. Antennal scrobes and preocular carinae absent. Antennae 11-segmented. Frontal lobes reduced, barely covering antennal insertions and diverging anteriorly. Frontal area subtriangular, distinct. In full-face view, posterior cephalic margin inflated laterally and strongly notched medially. Tubercles on mesosomal dorsum short, attenuate, and blunt. Metapleura with two spiniform processes between mid and hind coxae. Propodeum armed with a pair of short triangular spines. Node of petiole high, well-developed. Gaster lacking carinae or tubercles. In lateral view, pygidium rounded, laterally overlapping and concealing the hypopygium; in ventral view, pygidium posteromedially emarginate (i.e., V–shaped), the triangular hypopygium fitting within the emargination of the pygidium.

#### Gyne

Preocular carina absent. Mandible 4-toothed, apical tooth nearly twice as long as preapical tooth. Parapsidal lines inconspicuous.

#### Male

Mandibles broadly triangular with apical and subapical teeth present. Anterior margin of clypeus (clypeal apron) convex, projecting over mandibles, and with a long median seta. Discal cell present in forewing.

#### Etymology

*Cyatta* is a neologism constructed in part from the Brazilian Tupi language word *Cy*, meaning "sister," referring to its status, along with the genus *Kalathomyrmex*, as the sister clade to the remaining genera of the informal clade Neoattini, to which the genus *Atta*, the most conspicuous member of the Neoattini, belongs. 

### Cyatta abscondita, sp. n

urn:lsid:zoobank.org:act:3260572C-B7CE-429C-9440-27613BDBE69E 

**Figure 1:**
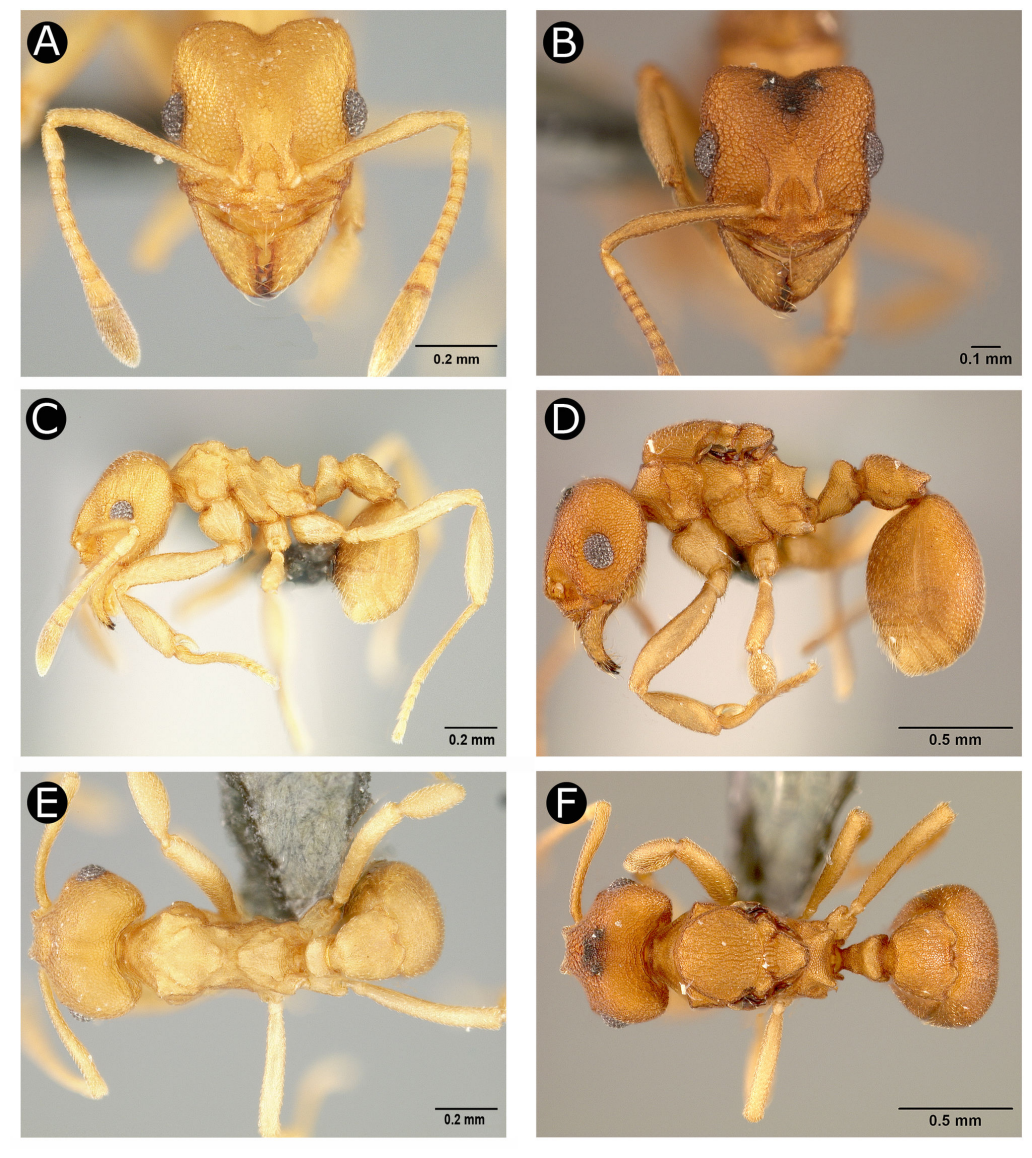
Holotype worker (USNMENT00758173) (A, C, E) and *Paratype* gyne (USNMENT00758174) (B, D, F) of *Cyatta abscondita*. (A, B) Full-face view. (C, D) Lateral view. (E, F) Dorsal view.

#### Holotype, worker

labeled: “BRAZIL: DF: Brasília; Faz. Água Limpa; 1106 m; 47.90133° W 15.9524° S ±5m; 20.ix.2011; (J. Sosa-Calvo*,* T.R. Schultz*,* C.T. *Lopes*); nest series; Cerrado *sensu stricto*; in ground; JSC110920-01” [MZSP, unique specimen identifier No. USNMENT00758173]. 

#### Paratypes

same data as holotype [3w, MZSP, USNMENT00758172, 00756921, 00758307], [1dg, 4w, USNM, USNMENT 00758174, 00758223, 00758316–18] [3w, USNM, USNMENT00521881 (EtOH vial)]. Same data as holotype, but “19.ix.2011; JSC110919-02” [1dg, MZSP, USNMENT00758175], [2w, USNM, USNMENT00758176–77] [1w, USNM, USNMENT00521910 (EtOH vial)]. Same data as holotype, but “16.iv.2010; JSC100416-04” [1w, MZC, USNMENT00758180] [1w, USNM, USNMENT00521908 (EtOH vial)]. Same data as holotype, but “1099 m; 47.90129° W 15.95242° S ±3m; 23.ii.2009; (*J Sosa-Calvo & TR Schultz*); nest series on ground; JSC090223-26” [1w, USNM, USNMENT00758178]. Same data as previous entry, but “16.iv.2010; nest series underground; JSC100416-01” [1w, DZUP, USNMENT00758319] [2w, 1dg (currently misplaced) USNM, USNMENT00758323–24, 00758325] [3w, USNM, USNMENT00521886 (EtOH vial)]. Same data as holotype, but “Garden near dorms; 1071 m; 47.93567° W 15.94938° S ±3m; 12.iv.2010; (*J Sosa-Calvo, TR Schultz, CT Lopes*); nest series; back yard; under ground; JSC100412-01” [1w, DZUP, USNMENT00758224], [1w, MZSP, USNMENT00758179], [3w, USNM, USNMENT00758320–22] [3w, USNM, USNMENT00521917 (EtOH vial)]. 

#### Worker measurements

Holotype (Paratypes, n=9). TL 2.40 (2.29–2.56), WL 0.62 (0.58–0.65), HL 0.53 (0.50–0.55), HW 0.48 (0.48–0.51), SL 0.48 (0.43–0.50) ML 0.34 (0.32–0.36), EL 0.13 (0.12–0.13), PL 0.17 (0.14–0.19), PPL 0.22 (0.20–0.24), GL 0.52 (0.49–0.64), CI 90 (90–95), SI 101 (90–100), MI 64 (63–68), FLD 0.17 (0.17–0.18).

#### Additional material examined

Same data as holotype, but “1099 m; 47.90129° W 15.95242° S ±3m; 16.iv.2010; (*J Sosa-Calvo & TR Schultz*); stray worker; JSC100416-08” [1w, USNM, USNMENT00521890 (EtOH vial)]. Same data as previous entry, but “15.iv.2010; (*J Sosa-Calvo & TR Schultz*); nest entrance; JSC100415-03” [4w, USNM, USNMENT00521907 (EtOH vial)]. **BRAZIL:** CE: Crateús; Croatá; RPPN Serra das Almas; [330m; 40.67978° W 05.16479° S]; 20–30.iv.2003; (*Y Quinet*); pitfall trap; Caatinga, *Carrasco*; SP 60 [1w, MZSP]. **DF**: Brasília; Reserva Ecol. IBGE; Parcela Bienal Tardia (Projeto Fogo); 1100m; 48.05000° W 15.85000° S; 30.i.2008; (*J Maravalhas*); pitfall trap;. Cerrado *sensu stricto*; B2/CT [2w, MZSP]. **MG**: Paineiras; Fazenda Olho D’Água; [611m; 45.53527° W 18.90628° S]; 22–24.v.1999; (*AA Tavares*); Winkler #2; Cerrado [1w, MZSP]. **MG**: Paracatu; Reserva Particular de Patrimônio Natural do Acangau; 671m; 47.0583056° W 17.1790833° S; 12.iv.2011; (*TLM Frizzo*); pitfall trap; Cerrado *sensu stricto* [2w, MBC–UFU]. **SP**: Itirapina; 9.5 km northwest of Itirapina; Broa preserve; 530m; 47.87754° W 22.18517° S; 21.vii.2011; (*C Rabeling & M Bacci Jr*); nest series; Cerrado *sensu stricto*; under ground; CR110721-04 [1w, MZSP, USNMENT00758220; 1m, MZSP, USNMENT00758204], [1w, MZC, USNMENT00758221], [2w, USNM, USNMENT00758219, USNMENT00758222], [6w, BLME & CRC, USNMENT00758190–95]. Same data, but CR110721-05 [1w, MZSP, USNMENT00758217], [1w, MZC, USNMENT00758218], [1w, USNM, USNMENT00758206], [10w, BLME & CRC, USNMENT00758207, USNMENT00758253–61]. Same data, but CR110721-08 [3w, BLME & CRC, USNMENT00758196–98]. Same data, but 26.vii.2011, CR110726-09 [3w, BLME & CRC, USNMENT00758199–201]. Same data, but 28.vii.2011, CR110728-01 [3w, BLME & CRC, USNMENT00758262-65], [1w, USNM, USNMENT00758205]. **SP**: Pindorama; Fazenda Águas Claras; 494m; 48.6873° W 21.4023° S; 16.viii.2011; (*GA Castilho*); Floresta Estacional Semidecidual; pitfall B1 [1w, MZSP]. **SP**: Sales; Estação Experimental; 414m; 49.3013° W 21.5222° S; 17.viii.2011; (*GA Castilho*); Floresta Estacional Semidecidual; pitfall I4 [1w, MZSP].

#### Measurements

TL  2.03–2.60, WL 0.52–0.66, HL 0.48–0.55, HW 0.46–0.54, SL 0.42–0.51, ML 0.20–0.38, EL 0.10–0.14, PL 0.13–0.19, PPL 0.21–0.25, GL 0.47–0.59, CI 87–95, SI 91–104, MI 40–68, FLD 0.16–0.18 (n=13).

#### Etymology

The specific name "*abscondita*" refers to the exceedingly secretive nature of this species, which, after being recognized from a few rare specimens, proved frustratingly elusive during multiple attempts to locate it in the field.

#### Worker. *Head*

**Figure 2:**
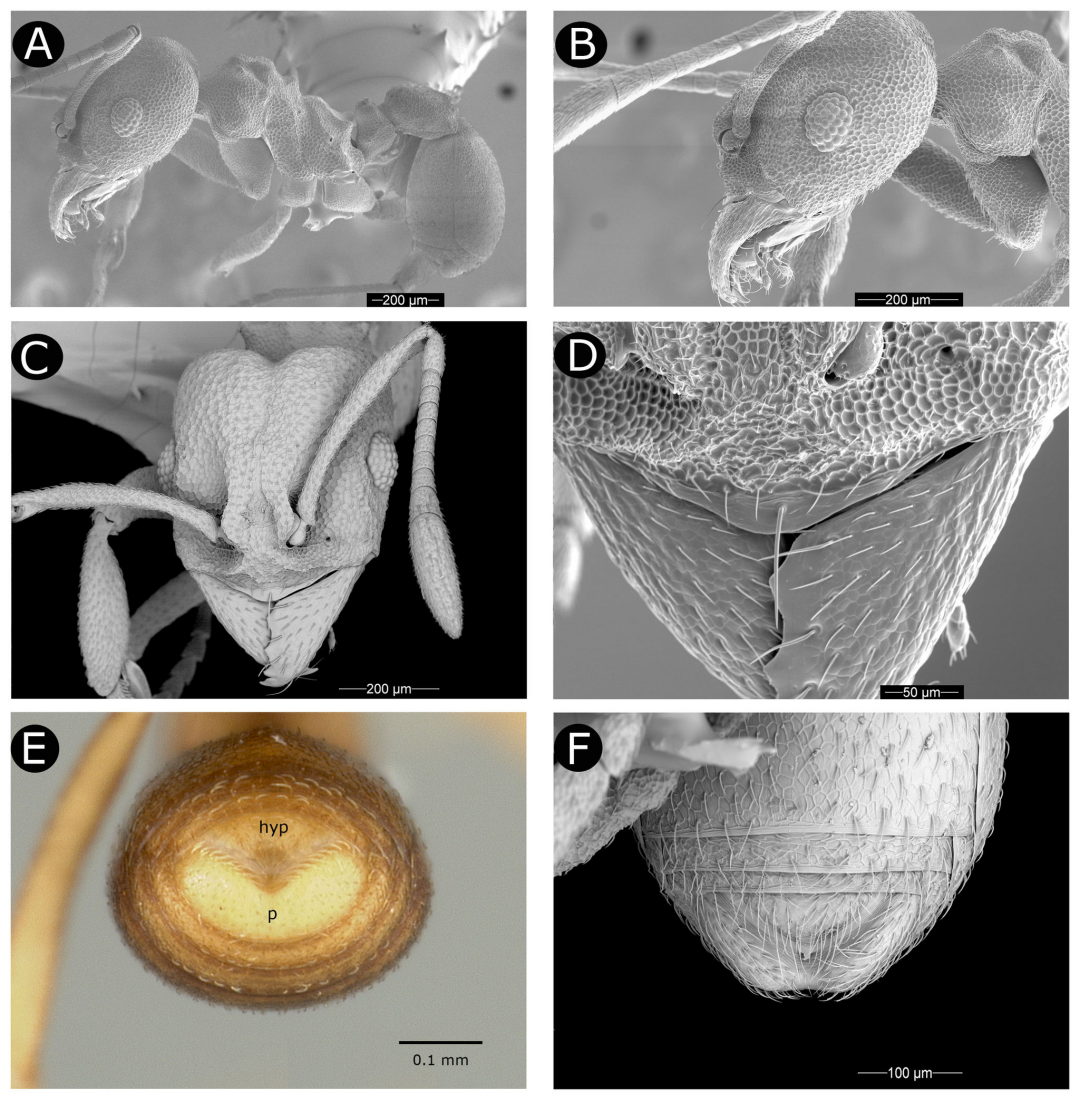
*Paratype* worker (USNMENT00758223) of *Cyatta abscondita*. (A) Habitus, lateral view. (B) Head, lateral view, indicating palp formula. (C) Head, full-face view. (D) Clypeal apron indicating origin of median unpaired seta. (E) Metasoma, posterior view. Pygidium (p) ‘V’-shaped, hypopygium (hyp) triangular. (F) Metasoma, ventral view, showing the median emargination of the pygidum and the triangular shape of the hypopygium.

in full-face view subrectangular, slightly longer than wide (CI 87–95); sides subparallel. Mandible subtriangular with four well-developed teeth; apical tooth twice as large as subapical tooth; diastema between subapical tooth and 3^rd^ tooth shorter or slightly shorter than diastema between 3^rd^ and 4^th^ teeth (Figure 2c); dorsum of mandible reticulate and with appressed hairs (Figure 2d); masticatory margin of mandible, including apical tooth, smooth, shining, and darker in color than rest of head, with long, simple hairs. Clypeal apron (anteclypeus) convex to almost triangular, smooth, and shining; unpaired median setae (length 0.07–0.10 mm) originating slightly before (anterior to) posterior edge of clypeal apron and almost or as long as antennal pedicel, not reaching apex of mandible (Figures 2c,d); clypeus with pair of lateral transverse carinae, each extending from below frontal lobe to mandibular insertion. Medially these carinae developed into lamellae perpendicular to clypeal face, thus forming a wall that divides the clypeus laterally into anterior and posterior areas, very likely homologous with clypeal morphology of closest relative, *Kalathomyrmex emeryi* (Figure 2c); medially clypeus is not so divided, face extending posterad between frontal lobes. Frontal lobes reduced, convex, barely covering antennal insertions (Figures 1a, 2c). Frontal carina fading out posteriorly at midlength of compound eye (Figures 1a, 2c). Well marked triangular area with concave anterior margin between frontal lobes reticulate, bordered anteriorly by rounded finger of clypeus, which extends broadly posterad between frontal lobes. Compound eye set slightly before middle of head, with 7–9 ommatidia at maximum length and 6 ommatidia at maximum width (33–47 ommatidia in total). Antennal scape covered with minute, simple, appressed hairs; antennal scape wider at seven-tenths of its length, and slightly surpassing posterolateral corners of head when laid back over head capsule; first funicular segment (pedicel) slightly longer than or as long as second and third funicular segments combined. In full-face view, cephalic margin deeply notched medially (i.e., at vertex) and rounded laterally (Figure 1a), shallow vertexal sulcus extending medially towards frontal lobes, fading at eye level; in lateral view, ventral face of head slightly convex. Hypostomal teeth absent. Palp formula 4,2 (Figures 2a,b).

#### Mesosoma

Profile of promesonotal dorsum in lateral view distinctly tuberculate, tubercles attenuate and blunt (Figures 1c,e, 2a). In dorsal view, promesonotum with raised shield-like area, broad anteriorly and narrowing posteriorly, distinctly separated from lower, lateral promesonotum; raised area formed anteriorly by triangular lateral pronotal tubercles and two median low and approximate pronotal tubercles and posteriorly by eroded remnants of promesonotal tubercles; lower, lateral area of promesonotum in dorsal view subtriangular, broader and anterolaterally angled anteriorly; in lateral view, inferior corner of pronotum rounded, lacking spines or angles. Anepisternum indistinctly separated from katepisternum. Metanotal groove relatively broad and strongly impressed, in lateral view extending to antero-ventral margin of metapleuron. Metapleura ventrally with two spiniform processes between mid and hind coxae, best seen by removing hind legs. Basal (dorsal) face of propodeum in lateral view a low, rounded, protuberance posterior to metanotal groove; in dorsal view, basal face very small, raised above remainder of propodeum, and narrowing anteriorly; declivous face of propodeum behind protuberance concave; propodeal spines triangular (Figure 1c), obliquely directed upwards and strongly diverging in dorsal view; declivity of propodeum much longer than base (dorsum); propodeal spiracle opening in an angle of 45° in relation to main body axis; in lateral view, propodeal lobes rounded without posterior projections. 

Peduncle of petiole vestigial; in lateral view, petiolar node well developed, subquadrate, with anterior face almost straight and vertical; dorsum of petiolar node short and almost flat, meeting vertical posterior face in slightly rounded angle; ventral face of petiole slightly concave or straight medially, lacking petiolar process (Figures 1c,e); in fronto-dorsal view, node of petiole shallowly V shaped, dividing node into a pair of rounded tubercles. Postpetiole robust, almost twice as long as petiole and slightly less than 0.5x gaster length; in dorsal view, postpetiole subtriangular, anterior margin rounded, lateral margins slightly diverging posteriorly; posterior margin with deep median impression, forming two distinct small lobes; in lateral view, anterior portion of postpetiole convex, postpetiole relatively compressed dorsoventrally; ventral projections absent (Figures 1c,e). In profile, gaster elliptical and dorsally finely reticulo-striate; in dorsal view, apical margin of pygidium (gastral segment IV, i.e., abdominal segment A7) medially emarginate, bilobed; gastral sternite IV (hypopygium, i.e., A7) covered with simple decumbent hairs; in lateral view, pygidium rounded, laterally overlapping and concealing the hypopygium; pygidium weakly reticulate and shiny; in ventral view, pygidium posteromedially emarginate (i.e., V–shaped), the triangular hypopygium fitting within the emargination of the pygidium (Figures 2e,f). Sting apparatus present, protruding through emargination on pigydium.

Color pale yellow to light brown; antennae, mandibles, and legs lighter than rest of body. Body integument areolate, with short appressed simple hairs, appearing almost hairless. 

 Gyne. As in worker description, but with caste-specific morphological differences as follows. All gynes studied are dealate. *Head*: Eyes large, with 10–11 ommatidia in maximum length and 9 ommatidia in maximum width, ~65 ommatidia total; median ocellus rounded, located in a median sulcus extending almost from the occipital carina in the back of the head to the middle of the frons; integument surrounding ocelli darker in color than elsewhere. Clypeus with unpaired median seta arising on short transverse wrinkle-like ridge that crosses clypeal apron; two to four short simple appressed hairs on clypeal apron on each side of median clypeal seta. In full-face view, cephalic border with median (vertexal) notch, not as deep as in worker. Mandibles dorsally coarsely rugose. 

#### Mesosoma

Pronotal dorsum conspicuously areolate, lacking anterior pronotal tubercles; lateral pronotal tubercles present, blunt and small; humeral pronotal tubercles vestigial. Mesoscutum, in dorsal view, rounded to slightly ovate and overall reticulo-rugose; dorsum of mesoscutum, in profile, almost flat; mesoscutal sulcus, in dorsal view, short, not extending more than 1/4 length of mesoscutum; notauli absent; parapsidal lines short, inconspicuous, and extending nearly to lateral margin of mesoscutum; transscutal suture conspicuous. Scuto-scutellar sulcus deep and with ~7 transverse carina; margin of axilla rounded, dorsally reticulo-rugose. Scutellum posteriorly weakly bidentate, dorsally rugose and with a shallow median longitudinal groove. Anapleural sulcus deep, with transverse carinae, dividing mesopleuron into anepisternum and katepisternum. Metanotal groove extended into a complete metanotal-propodeal suture (sensu Serna & Mackay [90]). In profile, metanepisternun (sensu Serna & Mackay [90] present, small. In profile, metanotal groove conspicuous, continuous with mesometapleural suture. Ventral metapleural processes present as a pair of spiniform tubercles between the mid and hind coxae, similar to, but longer than, those present in worker. Propodeum with pair of short, right-angled denticles; dorsum, lateral margin, and declivity of propodeum reticulate. *Metasoma*: petiole as in worker. Dorsum of postpetiole reticulo-rugose. Dorsum of gastral tergite IV (A7) rugulose; pilosity as in worker; gastral tergite and sternite IV (pygidium and hypopygium; A7) as in worker. 

#### Measurements

TL  3.27–3.32, WL 0.86–0.87, HL 0.65–0.66, HW 0.60–0.63, SL 0.56, ML 0.41–0.42, EL 0.16–0.17, PL 0.25–0.28, PPL 0.29–0.30, GL 0.79–0.81, CI 91–96, SI 89–93, MI 62–65, FLD 0.20–0.21 (n=2).

**Figure 3:**
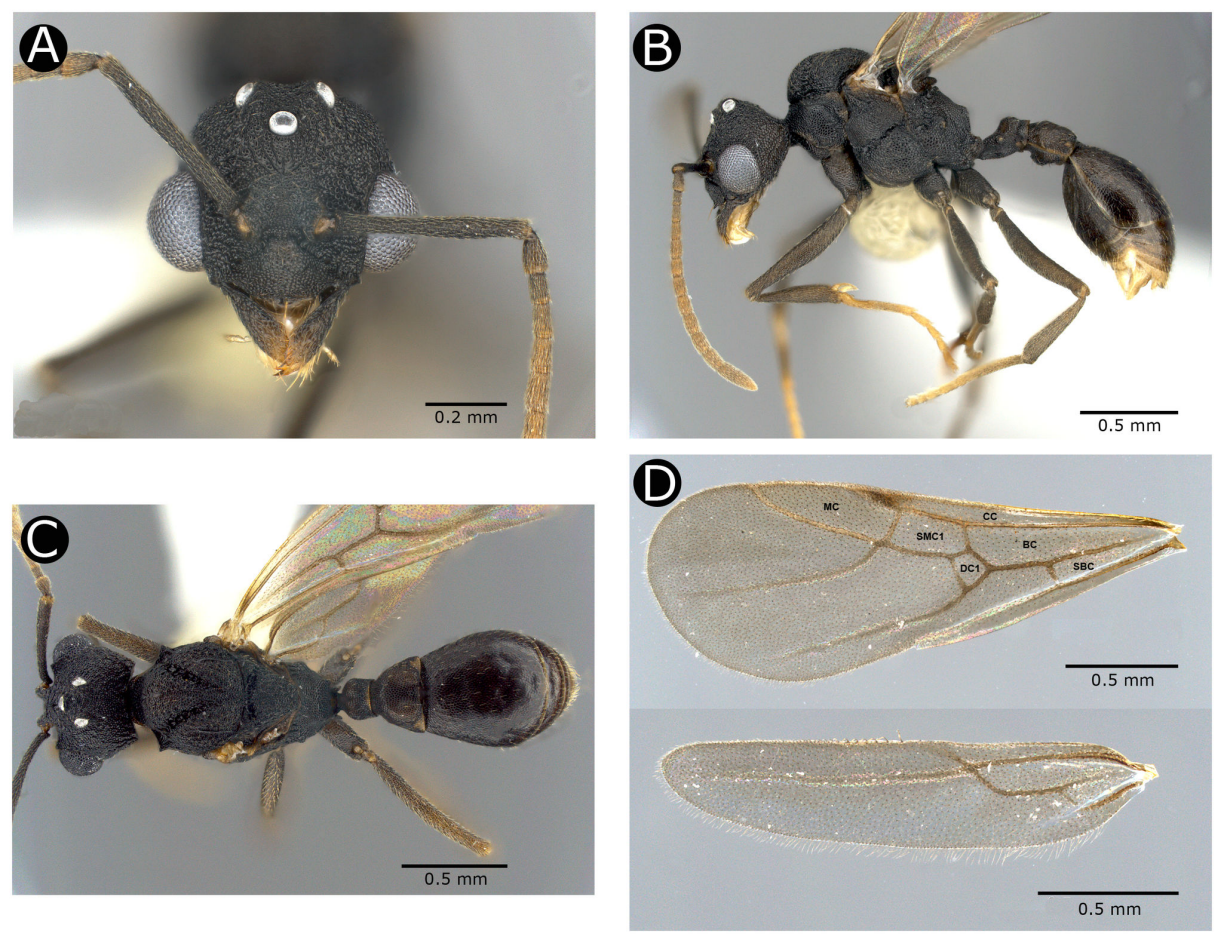
Male of *Cyatta abscondita* (USNMENT00758204). (A) Full-face view. (B) Lateral view. (C) Dorsal view. (D) Wings: forewing (top), hindwing (bottom). Cells: basal (BC), costal (CC), submarginal (SMC1), marginal (MC), subbasal (SBC), and discal (DC1).

Male. A medium-sized male with head large relative to size of the mesosoma. Mandibles broadly triangular, apical and subapical teeth present large; remaining tooth minute, indistinct; texture coarsely granulate. Palp formula 4,2. Clypeus broadly trapezoidal in frontal view; anterior margin convex, with a long median seta (0.11 mm) originating at the anterior margin and projecting over the mandibles; in lateral view anterior margin of clypeus forming a lamella projecting over the mandibles. Frontal lobes triangular, only partly covering the condylar bulbs of the scape in full face view. Antennae with 13 segments; scape surpassing the posterior border of the head by 1/3 of its length. Antennal funicular segment II (0.08 mm) almost as long as funicular segment I (pedicel; 0.11 mm) (Figures 3a,b). Eyes conspicuously large, at maximum diameter approximately half as long as the entire head, counting ~15 ommatidia in maximum width and ~23 ommatidia in maximum length. Ocelli large, elevated above the remainder of the head. Surface of head coarsely granulate, finely rugulose around the ocelli. Tergum of promesonotum not distinctly enlarged, giving the mesosoma a rather slender appearance in lateral view. In dorsal view, lateral pronotal teeth pyramidal, twice as wide at the base than high, with sharp tips. Propodeal spines reduced to broad teeth with rounded tips. Anterior peduncle of the petiole about the same length as the petiolar node. Postpetiole wider than long; trapezoidal in dorsal view; posterior margin slightly concave. In lateral view, postpetiole with a broadly rounded ventral lobe. Reticulate sculpture on gaster finer in appearance than on the remainder of body, which tends to be areolate; gastric tergites moderately lustrous; rest of body with a weak silky shine. Body surface sparsely covered with short appressed setae, only ventral side of postpetiole with 10 erect setae. Body dark, blackish brown; legs and antennae slight lighter in color, yellowish to dark brown. Forewings with closed basal (BC), costal (CC), submarginal (SMC1), marginal (MC), and subbasal (SBC) cells; submarginal cell 2 and discal cell 2 open (Figure 3d). Hindwings with closed basal cell and open marginal subbasal and discal cells (Figure 3d). Left forewing with closed discal (DC1) cell, whereas the right forewing lacking this cell. The presence of a closed discal cell in the forewing is, so far as is known to us, unique in the Attini; this character is absent in all other attine genera, including the closest relative of *C. abscondita*, *Kalathomyrmex emeryi*. A closed discal cell is plesiomorphically present in many ant species, including those in genera closely related to the Attini such as the Blepharidattini, Cephalotini, Dacetonini, and Pheidolini.

#### Measurements

EL 0.25, EW 0.27, FL 0.81, FLD 0.21, GL 0.87, HL 0.58, HW 0.73, IOD 0.42, ML 0.21, MI 37, PL 0.27, PPL 0.25, PPW 0.38, PrW 0.54, PW 0.21, SL 0.54, TL 3.21, WL 1.04, CI 126, SI 127 (n=1). 

**Figure 4:**
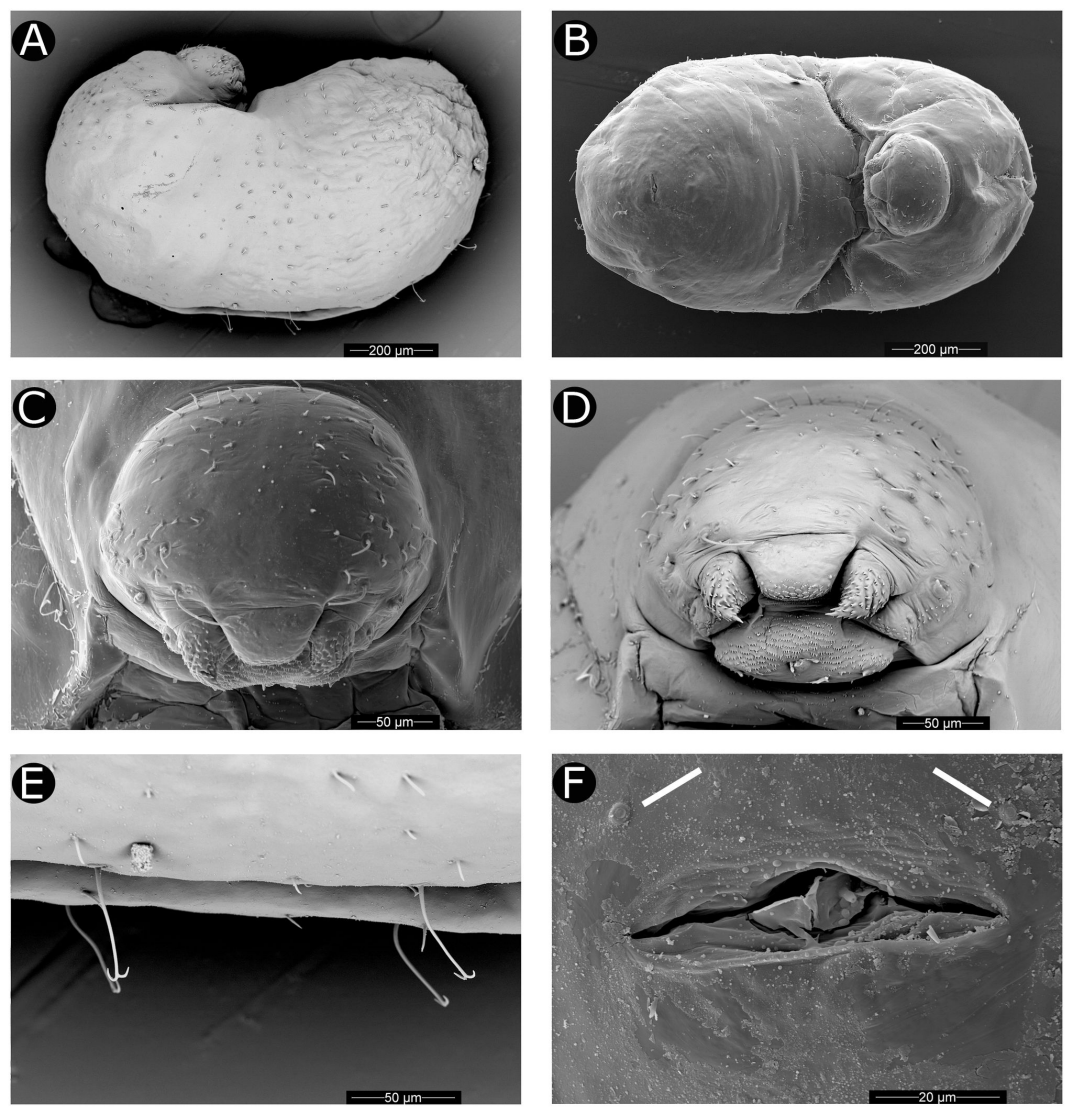
Larva of *Cyatta abscondita*. (A) Lateral view. (B) Ventral view. (C) Head, dorsal view. (D) Head, anteroventral view. (E) Anchor-tipped hairs on dorsum. (F) Anal region (white arrows indicate setal sockets; ventral at top).

Larva. Description based on SEM study of two specimens, late- (probably fourth) instar larvae of uncertain (but probably worker) caste. Due to collapsed condition of specimens, habitus profile could not be characterized with certainty, but is consistent with the "attoid" profile category of Wheeler & Wheeler [104], i.e., with a moderately curved, ventrally shortened profile. Thoracic-abdominal articulation apparently absent, thoracic intersegmental constrictions superficial, deep lateral depressions associated with abdominal spiracles absent, all states shared with other Attini. Remarkably, body hairs present dorsally and laterally, a condition otherwise common in the Myrmicinae but rare in the Attini, in which larvae usually lack dorsal and lateral hairs. In the Attini, such hairs are known to be present only in the larvae of *Mycocepurus goeldii* and *M. smithii* [92], where their presence may be plesiomorphic, and in *Sericomyrmex* and some *Acromyrmex* species, where their presence is likely secondarily derived. Predominant hair type bifurcate with "anchor tips" (Figures 4a,d). Two rows of dorsomedian, very long anchor-tipped hairs present (Figure 4e). Labrum monolobate, narrow, bulging. Anterior setae present as papillae. Mandibles typically attine: short, fleshy, subconical. A distinct, undivided apical mandibular tooth and no subapical teeth; spinules evenly distributed on all mandibular surfaces. Mandibular gnathobases absent. Basal portions of maxillae fused with head capsule. Maxillary palp widely removed laterad from galea, a synapomorphy for the Neoattini. Galea reduced, present as two sensilla surmounting a low protuberance, as in all Attini except for some *Myrmicocrypta* species. Maxillary palp digitiform, maxillary accessory palpal sensillum absent. A single seta present laterad of maxillary palp, a character shared with *Mycocepurus* species. As in most attines, labium feebly protruding, lateral sericteral protuberances absent, labial palps digitiform. Labial spinules present on anterior surface slightly dorsal to the sericteries. Hypopharyngeal spinules multidentate and apparently densely distributed. On the head, genal lobes absent, a state in the Attini shared with *Myrmicocrypta*, *Apterostigma*, *Mycocepurus*, and *Mycetarotes* species. Supra-antennal setae present and abundant, a condition common in the subfamily but otherwise present in the lower Attini only in *M. goeldii*. Subantennal (genal) setal arrangement plesiomorphic for the tribe, consisting of around 12 setae on each gena. Supraclypeal setae present and setiform. Two clypeal setae present. Spinules absent on the head dorsad of the labrum, the state common to most attines. Due to the poor condition of specimens, most ventral thoracic/abdominal characters could not be studied, including the presence/absence of: leg vestiges, prothoracic food anchor, ventromedian protuberances on various segments, papilliform spinules, and hairs. 

Two setal sockets occur ventral of the anal opening on abdominal segment IX (Figure 4f). No other setae are associated with the anal opening. Ventral anal lip absent.

#### Comments

The new genus *Cyatta* shares with other genera belonging to the tribe Attini: (i) the presence of a thick unpaired median seta arising from the clypeal apron (considered a synapomorphy for the tribe by Brandão & Mayhé-Nunes [105]; but, along with the clypeal apron, presumed secondarily lost in *Kalathomyrmex* [30]); (ii) 11 antennal segments in the worker and gyne, 13 in the male (the latter secondarily reduced in some *Cyphomyrmex*, *Mycetagroicus*, *Sericomyrmex*, *Trachymyrmex*, and social parasites); (iii) palpal formula 4,2 (plesiomorphic for the Attini, secondarily reduced in *Apterostigma* and some social parasites). Larvae of *Cyatta* share with the larvae of other attine genera the: (iv) thoracic-abdominal articulation apparently absent; (v) thoracic intersegmental constrictions superficial; (vi) deep lateral depressions associated with abdominal spiracles absent; (vii) short, narrow labrum; and (viii) fleshy, subconical mandibles. Behaviorally, *Cyatta* shares with other Attini the cultivation of fungi for food. 

The genus *Cyatta* shares with other members of the neoattine clade: (i) the antennal scape of the male long, longer than the sum of the length of antennal funicular segments I–III; (ii) the first funicular segment (pedicel) of the antenna of the male longer than second funicular segment; (iii) the petiole in workers somewhat sessile; (iv) the lack of hypostomal teeth in workers and gynes; and (v) the maxillary palp of the larva widely removed laterad from the galea.

*Cyatta* shares with its sister genus, *Kalathomyrmex*, (i) the lack of a tubercle or spine on the inferior lateral margin of the pronotum, a symplesiomorphy shared with the paleoattine clade; (ii) the clypeus with a pair of lateral transverse carinae, each extending from the frontal lobe to the mandibular insertion and each medially developed into a lamella perpendicular to the clypeal face, thus forming a wall that divides the clypeus laterally into anterior and posterior areas; and (iii) the mandibles of the male with three teeth, of which the apical and preapical teeth are the largest and have a multidentate (saw-like) margin.

*Cyatta* differs from its sister genus *Kalathomyrmex*, however, by (i) having, on the forewing of the male (forewing of gyne unknown), a closed marginal cell (Figure 3d) (open in the forewings of both the male and gyne of *Kalathomyrmex* [Klingenberg and Brandão ([30]), therein as radial cell]); (ii) the mesoscutum of the male with strongly impressed notauli (absent in the male of *Kalathomyrmex*); (iii) the pronotum of the male with lateral pronotal tubercles present, pyramidal (the pronotum in the male of *Kalathomyrmex* lacks any tubercles); and (iv) the psammophore absent in the worker, the gyne, and the male.

In addition to the previously mentioned absence of an inferior pronotal tubercle in adult workers, shared with *Kalathomyrmex*, *Cyatta* differs from all or most other Neoattini in a number of larval character states shared with the Paleoattini, suggesting that they may be retained symplesiomorphies, including: (i) dorsal and lateral body hairs present and abundant, shared with *Mycocepurus* species; (ii) a single seta present laterad of the maxillary palp, shared with *Mycocepurus* species; (iii) supra-antennal setae present and abundant, shared with *Mycocepurus goeldii*; (iv) genal lobes absent, shared with the paleoattines and the neoattine genus *Mycetarotes*. Larval characters are unstudied in *Kalathomyrmex*. In addition, the worker and gyne of *Cyatta* differ from members of the neoattine clade by (v) the node of the petiole well developed, high (Figures 1c,d, 2a).

Most notably, *Cyatta* differs from all other attine genera and species by the following autapomorphies: (i) mandible of the worker and gyne with four teeth (Figures 1b, 2c); (ii) in ventral view, metapleura of the worker and gyne with two spiniform processes between the mid and hind coxae, apparently absent in the male; (iii) apical margin of the pygidium medially emarginate, V-shaped (Figures 2e,f); and (iv) forewing of the male with a closed discal cell (Figure 3d). 

Based on the extreme degree of morphological divergence and the results of the divergence dating analyses (see below), we have chosen to describe *Cyatta* as a new genus rather than to describe it as a species within the genus *Kalathomyrmex*.

#### Discovery history

In 2003, a single stray worker of *C. abscondita* was taken in a pitfall trap as part of an ant survey conducted at the Reserva Particular do Patrimônio Natural Serra das Almas, Crateús, CE, Brazil, a relatively undisturbed area of Caatinga, a biome characterized by deciduous thorny woodland vegetation [106]. The specimen was deposited in the MZSP ant collection, where it was at first associated with the *Mycetophylax* species group, but subsequently recognized as a new neoattine genus by CK and CRFB. This isolated specimen inspired the first attempt to locate *C. abscondita* in the field in Serra das Almas in 2009 by CRFB and RMF. Unfortunately, it was the end of the rainy season and the soil was covered by a dense layer of grass, impairing observations of all small and inconspicuous ants. Visual searching and leaf-litter extraction failed to locate additional specimens, as did subsequent surveys at the same locality.

In 2008, two workers were taken in pitfall traps in the Instituto Brasileiro de Geografia e Estatística (IBGE) Cerrado preserve, near FAL in Brasília, DF, Brazil. These specimens, deposited in the MZSP, inspired attempts by JSC, TRS, CTL, and HLV to locate the species at this locality beginning in 2009. The first such attempt yielded only the collection of a series of stray workers and an unsuccessful nest excavation; however, subsequent visits resulted in the excavations of multiple nests and collections of gynes, larvae, and cultivated fungi. 

The only known male of the species was fortuitously collected in 2011 by CR and MB when they accidentally encountered two nests of *C. abscondita* while excavating a nest of *Mycocepurus goeldii* in the Broa Preserve, Itirapina, SP, Brazil.

The earliest known collection of *C. abscondita* was that of a stray worker taken in a leaf-litter sample in Paineiras, MG, in 1999, only recently discovered in the entomological collection at MZSP and recognized as belonging to this species. Most recently, in 2011, two workers of *C. abscondita* were recovered from pitfall traps in fragments of semideciduous forests in the Sales and Pindorama municipalities in northwestern São Paulo state. This history of discovery indicates that *C. abscondita* is rarely collected by traditional methods. The cryptic nature of foragers and of nest entrances makes it almost invisible to traditional hand collecting. The rarity of individuals in pitfall and leaf-litter samples remains puzzling, since the concentrations of nests encountered at FAL and Broa Preserve suggest that it is locally abundant. Now that the genus and species are recognized and described, we hope that additional specimens will be identified in unsorted material in collections as well as in newly collected material from ant surveys in Brazil and perhaps even elsewhere in South America.

### Natural history

### Macrohabitat

**Figure 5:**
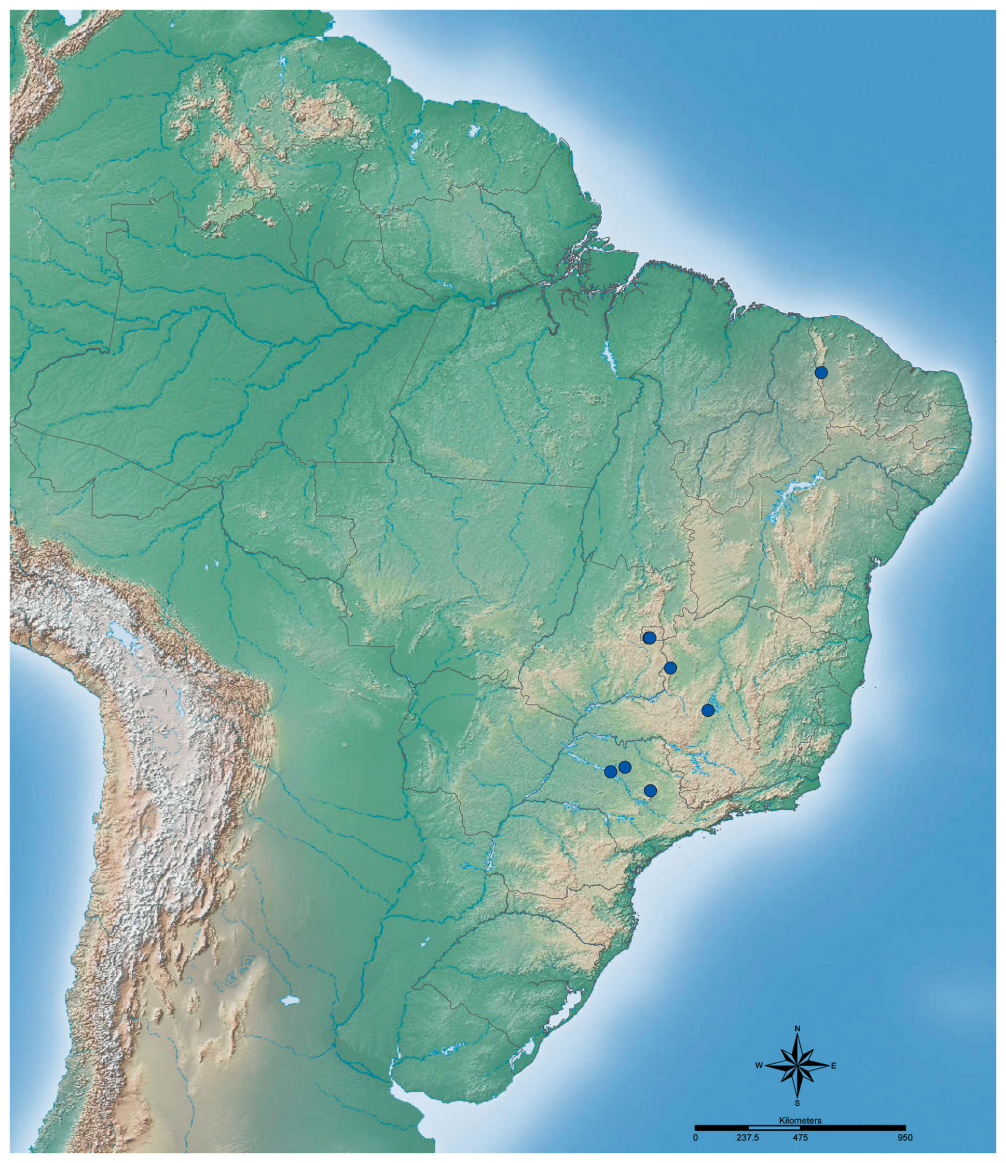
Known distribution of *Cyatta abscondita*.

Most collections of *C. abscondita* are from Cerrado localities (Figure 5). These include Fazenda Água Limpa (FAL) near Brasília, the Broa preserve in São Paulo, the IBGE Cerrado preserve in Brasília, DF, the Fazenda Olho D’Água in Paineiras, MG, and the Reserva Particular do Patrimônio Natural (RPPN) do Acangau in Paracatu, MG, all of which are characterized by diverse Cerrado phytophysiognomies, ranging from campo limpo to Cerrado *sensu stricto* [107]. The predominant habitat, Cerrado *sensu stricto*, is a low canopy arboreal woodland that is characterized by the presence of small trees with a canopy height of less than 7 meters, shrubs, and abundant ground vegetation [108–110]. Cerrado soil is typically a red-yellow latosol, largely composed of well-drained and nutrient-poor quartz sand with moderate clay content below 15% [107,111]. Both FAL and Broa have typical Cerrado climates with a marked dry season from May to September and with a mean annual temperature and precipitation of 23°C and ~1420 mm, respectively [110]. For a complete account of vegetation and soil compositions at FAL and Broa, see [112] and [113]. The label data associated with the *C. abscondita* worker from Fazenda Olho D’Água indicates that it was obtained from a leaf-litter sample from a Winkler extractor. This suggests that this worker was likely taken in either a riparian forest or in “Cerradão,” because it is in such areas that trees are dense enough to produce conspicuous accumulations of leaf litter. 

Some specimens of *C. abscondita* have been taken outside the Cerrado biome (Figure 5). Most notably, a stray worker was recovered from a pitfall sample taken in 2003 in a relatively undisturbed area of Caatinga, a biome characterized by deciduous thorny woodland vegetation [106], in the RPPN Serra das Almas, Crateús, CE, Brazil. More recently, in 2011, two workers of *Cyatta abscondita* were taken in pitfall traps in fragments of semideciduous forests in northwestern São Paulo state. This region is considered a transition zone between Cerrado and endangered coastal Atlantic Forest [114]. 

#### Microhabitat

**Figure 6:**
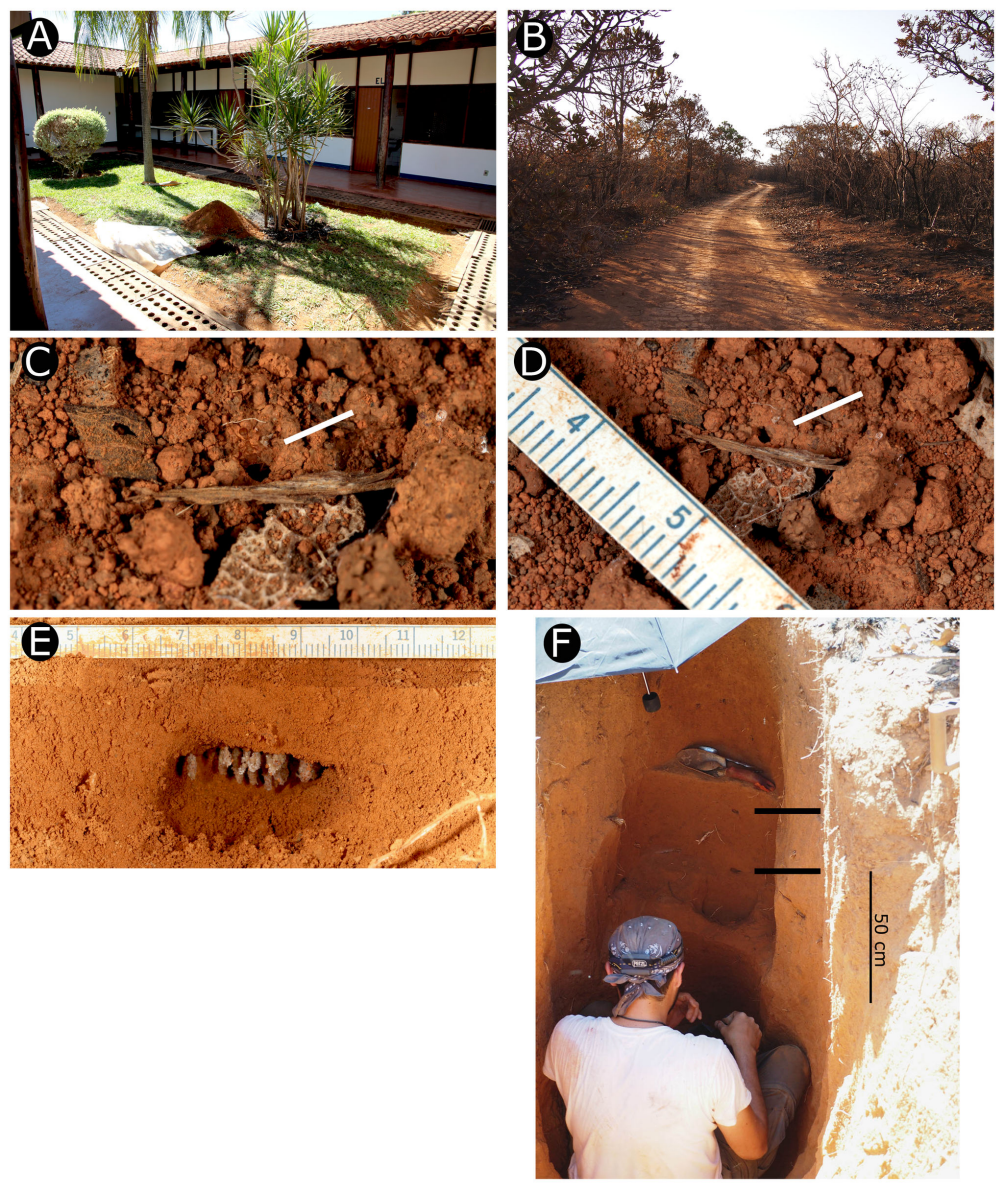
Habitat of *Cyatta abscondita*. (A, B) Fazenda Agua Limpa (FAZ). (A) Excavation of nest JSC100412-01 in dormitory garden area. (B) Cerrado *senso* *stricto*, where colonies were found on the side of the road. (C,D) Nest entrance of *Cyatta abscondita* (white arrows). (C) Worker entering nest. (D) Nest entrance of *Cyatta abscondita*, consisting of an inconspicuous ~1mm diameter hole in the ground. (E) Chamber with pendant fungus garden. (F) Excavation of nest 4 (JSC110920-01). Black bars indicate two chambers, the lower one 104 cm below the surface.

Four nests of *Cyatta abscondita* were excavated at FAL and two nests at Broa (summarized in Table 1). Nest entrances of four additional nests were located at FAL; however, attempted excavations of these nests failed. At FAL, seven of the excavated nests occurred on the side of a little-used dirt service road in Cerrado *sensu stricto* (Figure 6b) and the eighth (nest 1, Table 1) on the lawn of the FAL dormitories (Figure 6a). The roadside nests were directly exposed to sunlight for most of the day, whereas the lawn nest was shaded by the adjacent building in the morning and afternoon. At the Broa preserve, both nests occurred in the shade of trees in Cerrado *sensu stricto*. Both Broa colonies were excavated serendipitously during excavations of *Mycocepurus goeldii* nests and the *C. abscondita* nest entrances were not observed. 

#### Foraging behavior

Foraging workers of *Cyatta abscondita* are difficult to locate because colony sizes are small, workers forage individually, and individuals are very small and cryptic (Figure 6c). In mid-September, which coincides with the beginning of the rainy season at FAL, three to four individuals from three different nests, including nests 3 and 4 (Table 1), were observed foraging at night between 22h and 23h. In February and April, at the end of the rainy season, ants from five FAL nests, including nests 1 and 2 (Table 1), were likewise observed foraging individually only after sunset. Only in the case of FAL nest 1 were workers observed to forage in the early afternoon on two consecutive days in April between 13h and 15h during a time when the nest entrance was shaded from direct sunlight. Unlike the other nests, nest 1 occurred in a well-watered, human-managed grassy lawn. Nests 5 and 6 (Table 1), which were collected in July at the height of the dry season at the Broa preserve in São Paulo State, were located in the shade and, unfortunately, neither foraging nor nest-building activity was observed.

The entrance of one FAL nest (JSC090223-26) was located ~4.5 centimeters from the entrance of an adjacent *Mycocepurus goeldii* nest. At around 23h a *Cyatta abscondita* worker was observed lurking slightly inside the nest entrance while workers of *M. goeldii* foraged on bait (granules of Cream of Rice cereal) placed near the nest entrances. When *M. goeldii* workers were absent, the *C. abscondita* worker darted out to retrieve a piece of bait and quickly returned to its nest. This lurking and rapid foraging behavior was repeated until the supply of bait was depleted. In rare cases of contact between *C. abscondita* and *M. goeldii* workers, *C. abscondita* workers were observed to remain motionless. Aggressive interactions were not observed.

#### Nest architecture

At FAL, nest entrances of *Cyatta abscondita* consisted of a single, inconspicuous, hole in the ground of approximately 1 mm in diameter without any accompanying mound or turret (Figures 6c,d). As mentioned above, the entrance of one nest was located in the mound of a *Mycocepurus goeldii* colony ~4.5 cm from the *M. goeldii* nest entrance. At the Broa preserve, nest chambers of *C. abscondita* were encountered serendipitously while excavating *M. goeldii* nests and the nest entrances were not observed.

At FAL, excavations of eight nests were attempted. Four excavations (JSC090223-26, JSC110914-02, JSC100415-03, JSC100416-04) failed (i.e., neither chambers containing fungus gardens nor gynes were found, but workers were collected at their respective nest entrances), but chambers containing fungus gardens were located in four nests (Table 1). Nests contained three to eight chambers. In FAL nests 1 and 3, which contained 4 and 3 chambers respectively, chambers were roughly arranged vertically below the nest entrance (Table 1), although it is possible that additional, laterally dispersed chambers were missed during the excavations. At FAL, nests 2 and 4 contained 7 and 8 chambers, respectively, some of which occurred at the same depth but were laterally separated from each other (Figure 6f). The shallowest chamber encountered (nest 3, FAL) was 29 cm deep and the deepest chamber (nest 6, Broa) was 195 cm deep. Because no gynes were found during the nest excavation at Broa, it is entirely possible that additional chambers occurred below a depth of 2 meters. Chambers were elliptically shaped, 1–2.5 cm wide and 2–5 cm high (Figure 6e). The largest garden chamber encountered (nest 2, FAL) was 2.5 x 5.5 cm; at Broa, a similarly sized chamber (nest 5) contained ~50 hanging garden filaments (Figure 6e). Some chambers were empty; in one case, an empty chamber contained three polydesmid millipedes. 

#### Demography

Dealate gynes were collected in three of the eight excavated nests, suggesting that in five nests additional chambers remained undiscovered in the soil, or that gynes escaped into adjacent tunnels. In each of the three queenright nests (nests 2, 3, and 4), a single gyne was consistently encountered in the deepest chamber (see Table 1); however, additional chambers may have been present at greater depths, because excavations were generally terminated upon encountering the gyne. Brood was found only in FAL nest 4, which was collected in September, the beginning of the rainy season, suggesting that colonies of *Cyatta abscondita* reduce their reproductive activities during the dry season. The maximum number of workers encountered in colonies ranged from ~20 (FAL nest 4) to 26 (Broa nest 5). One male was collected in nest 5 at Broa on 21 July. 

#### Garden morphology

Gardens were pendant and arranged in filamentous curtains suspended from the chamber ceiling (Figure 5e), similar to the fungus gardens of *Mycocepurus* species [70,93,115,116] and of *Kalathomyrmex emeryi* (TRS, JSC, pers. obs.). Single fungal curtains were 5–6 mm long and 1–2 mm wide and a maximum number of 50 curtains were found in a single chamber. Curtains were directly attached to the soil of the chamber ceiling rather than to rootlets. In nest 4, which was maintained in laboratory culture for three months, workers attached garden filaments to the plastic ceiling of the nest box and cultivated suspended gardens. The filaments were firmly attached to the plastic ceiling by an unknown mechanism.

#### Phylogeny

**Figure 7:**
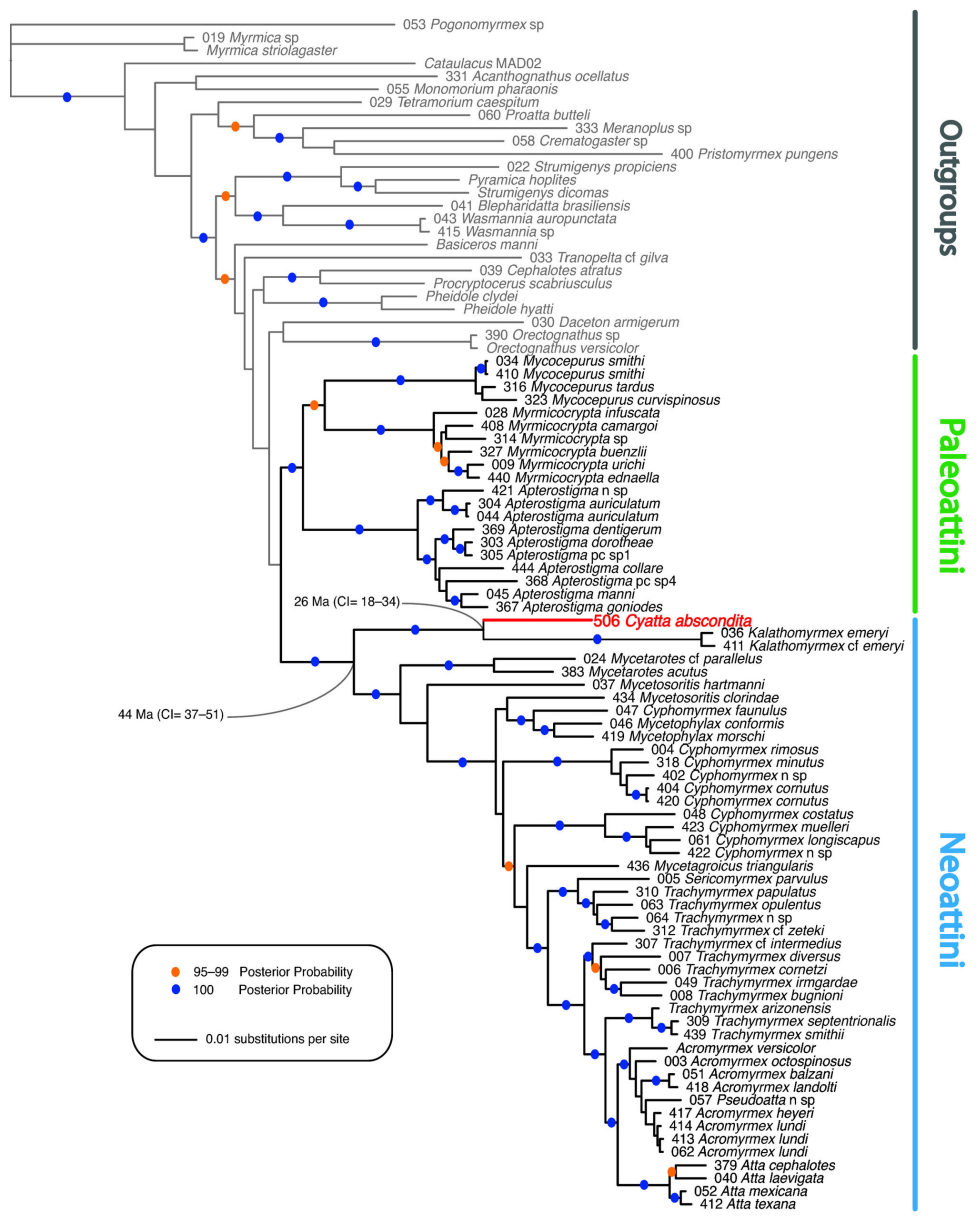
Phylogenetic position of *Cyatta abscondita*. This phylogeny of 66 fungus-farming and 26 outgroup ant species results from a Bayesian analysis of four nuclear protein-coding genes (see text for details). Fungus-farming ant species are indicated by bold black branches; the branch subtending *Cyatta abscondita* is indicated in red. Gray branches correspond to non-fungus-farming taxa. Blue dots on branches represent Bayesian posterior probabilities of 100; orange dots represent Bayesian posterior probabilities of 95–99. Divergence time of *Cyatta* and *Kalathomyrmex* estimated at 26 Ma (95% CI= 18–34) and divergence of the clade (*Cyatta* + *Kalathomyrmex*) from the rest of the Neoattini estimated at 44 Ma (95% CI= 37–51).

Results of molecular phylogenetic analyses incorporating four nuclear gene sequences from *Cyatta abscondita* confirm the previous finding [95] that the tribe Attini is divided by an ancient divergence into two major clades, the Paleoattini and the Neoattini (Figure 7). *Cyatta abscondita* occupies a relatively isolated position in the latter clade, distantly related to the monotypic genus *Kalathomyrmex* Klingenberg & Brandão, the result of an early divergence in the Neoattini. Its phylogenetic position, nested well within the paraphyletic group of "lower attine ants," strongly supports the hypothesis that *C. abscondita* practices "lower attine agriculture" [58]. 

A relaxed-clock-divergence dating analysis conducted in BEAST using the Bayesian uncorrelated lognormal approach with a normal prior on the root node (as described in Schultz & Brady [95]), resulted in a chronogram in which *Cyatta* occupies a position identical to that in the MrBayes results shown in Figure 7. The BEAST chronogram indicates that *Cyatta* and *Kalathomyrmex* diverged 26 Ma (95% CI= 18–34) and that the earliest possible divergence of the clade (*Cyatta* + *Kalathomyrmex*) from the rest of the Neoattini occurred 44 Ma (95% CI= 37–51).

## Discussion

The pursuit and discovery of phylogenetically informative new species are arguably among the most important enterprises in systematic biology. Numerous studies have demonstrated the significant effects of taxon representation on phylogenetic inference, including, in addition to tree topology, ancestral character state reconstruction, divergence time estimation, and inferences of evolutionary rates [117–119]. Ward et al. [118] showed that the exclusion of a single relict species resulted in the incorrect reconstruction of the phylogeny of the ant subfamily Dolichoderinae (Formicidae). In addition, the recently discovered ant genus *Martialis*, a relict subterranean species known from the Amazon forest in Brazil, has been shown to be the only hitherto known representative of an early diverging branch of the ant tree of life [27]. Within the Attini, the recently described ant genus *Mycetagroicus* [105,120] was found to be the sister group to the higher attine ants (*Sericomyrmex*, *Trachymyrmex*, *Acromyrmex*, and *Atta*) and thus to occupy a phylogenetic position transitional between lower and higher agriculture [95]; however, until very recently its fungal cultivar association remained unknown. Subsequent field work documented that *Mycetagroicus cerradensis* cultivates a lower attine fungus, suggesting that biological investigations of the genus are critical for understanding the evolutionary transition from lower to higher agriculture [94]. This strategy of reciprocal illumination (i.e., information gathered from the field informing phylogenies, and phylogenies guiding field work) plays a key role for reconstructing and understanding the macro- and micro-evolutionary processes driving the attine agricultural symbiosis. 

The results reported here indicate that *Cyatta abscondita* possesses an intriguing mosaic of characters, some that are shared with paleoattines, others that are shared with neoattines, and at least one that is shared only with non-attine ants. Because the Neoattini, Paleoattini, and the non-attine Myrmicinae span the ancestral node of the tribe Attini, this combination of character states suggests that the morphology, behavior, fungal associations, nest architecture, and other biological characters of *C. abscondita* are potentially informative about plesiomorphic character states within the tribe and, consequently, about the early evolution of ant agriculture.

At least one character of *C. abscondita*, the presence of a closed discal cell in the forewing of the male, is unknown in all other Attini. If, as we suspect, this is a retained plesiomorphy rather than an autoapomorphy, then the absence of the discal cell in other Attini must be the result of at least three parallel losses, one in the Paleoattini, one in the Neoattini, and one in *Kalathomyrmex*. 

Another character previously unknown in the Attini is the presence of at least two rows of elongate anchor-tipped hairs (Figures 4a,e) on the mid-dorsum of the larva. The function of such anchor-tipped hairs has recently been studied in the non-fungus-farming ant *Pheidole rhea*, which utilizes these specialized setae to hang fourth-instar larvae from the nest walls [121]. The widespread presence of this character state in non-fungus-farming ants strongly suggests that it is plesiomorphic for the Attini and that its presumably derived absence in most Attini may be connected to the fact that larvae are usually more or less enveloped in mycelium deep within the fungus garden rather than hung from the chamber wall. 

Presumed plesiomorphic adult character states shared by *C. abscondita* and the Paleoattini include the presence of a rounded inferior lateral margin of the pronotum, also retained in *Kalathomyrmex emeryi*. All other neoattine genera, from *Mycetarotes* to the leaf-cutter ants, have a denticle or tooth in this position. A number of larval character states are shared with species of the paleoattine genus *Mycocepurus*, including the presence of dorsal and lateral hairs, the presence of a single seta laterad of the maxillary palp, and abundant supra-antennal setae. The absence of genal lobes is shared with all Paleoattini as well as, in the Neoattini, with *Mycetarotes* species. (Larval characters of *Kalathomyrmex* have not yet been documented.) The striking pendant, curtain-like morphology of the fungus garden is a character state shared with *Mycocepurus* species as well as with *Kalathomyrmex*.

Presumed neoattine synapomorphies shared by *C. abscondita* and other Neoattini include, in adults, (i) the lack of hypostomal teeth, also secondarily lost in some species of *Apterostigma*; (ii) the antennal funicular segment II of males short, as long as or slightly longer than funicular segment I (pedicel), whereas in the males of the paleoattine genera the funicular segment II is long, almost twice as long than the pedicel; and (iii) the wide separation of the maxillary palp from the galea in the larvae.

Although both molecular and morphological data indicate that *Cyatta abscondita* is the sister species of *Kalathomyrmex emeryi*, it is a very distant sister, having diverged from their most recent common ancestor approximately 26 mya. As far as is currently known, *C. abscondita* shares with *Kalathomyrmex* two unique morphological characters, the form of the clypeus and the morphology of the mandibles in the male. In common with the paleoattines, but differing from all other neoattines, *C. abscondita* also shares with *Kalathomyrmex* a rounded inferior pronotal corner. In contrast to these shared character states, two synapomorphic and one symplesiomorphic, *C. abscondita* notably lacks the defining feature of *Kalathomyrmex*, the basket-like psammophore (Gr. *kalathos* = "basket") and differs not only from *Kalathomyrmex* but from all other Attini in a number of striking characters, including the 4-toothed mandible, the presence of paired ventral pleural spiniform processes, and the presence of a discal cell in the wing of the male. In fact, the morphology of *C. abscondita* is a mosaic of characters of the paleoattine and neoattine clades as well as of closely related non-attine myrmicines. For these reasons we choose to recognize *Cyatta abscondita* as a distinct genus within the fungus-farming ants.

The discovery, description, and mapping of biological diversity is essential for devising strategies for protecting biodiversity hotspots, i.e., areas that support high concentrations of endemic species and that are threatened due to the rapid loss of habitat as a result of years of unsustainable human exploitation [86], [122]. The Brazilian Cerrado is one such biodiversity hotspot. With only 20% of the original primary habitat remaining, and with only 6.2% of that habitat protected, the Brazilian Cerrado sustains more than four thousand species of endemic plants and more than a hundred species of endemic vertebrates [86]. The Caatinga is even less protected than the Cerrado [123]. The discovery of *Cyatta abscondita* in the Cerrado and in the poorly explored Caatinga habitats of Brazil suggests that increasing the search for cryptic and inconspicuous species will lead to discoveries that will fundamentally alter our understanding of insect evolutionary history.
